# Development, implementation, and evaluation of the effectiveness of an intervention program to improve the sexual competence of young adult women about to get married: a protocol study

**DOI:** 10.1186/s12978-024-01754-9

**Published:** 2024-02-16

**Authors:** Zahra Sadat Mousavi, Mojgan Janighorban, Mahnaz Noroozi

**Affiliations:** 1 Sexual and Reproductive Health, Student Research Committee, Isfahan University of Medical Sciences, Isfahan, Iran; 2 Reproductive Sciences and Sexual Health Research Center, Isfahan University of Medical Sciences, Isfahan, Iran; 3 Department of Midwifery and Reproductive Health, Isfahan University of Medical Sciences, Isfahan, Iran

**Keywords:** Sexual behavior, Sexual activity, Marriage, Young adult, Program, Protocol study

## Abstract

**Background:**

Having competence in initiating sexual interactions is one of the challenges of sexual health in any society. Given that the social, cultural, and religious background of some societies can prevent the acquisition of sexual competence in young women, this study will be done to design, implement, and determine the effectiveness of an intervention program to improve the sexual competence of young women on the eve of marriage.

**Methods:**

The current research is a mixed-method study in a qualitative-quantitative sequence. In the first phase, a qualitative study will be conducted to explore the needs of sexual competence in young adult women about to get married and ways to improve it. Then, after the literature review and combining it with the results of the qualitative study, a draft of the intervention program will be developed. After reviewing the content of the program and validating it in the panel of experts, the final program will be developed. In the second phase, the effect of the program to promote the sexual competence of adult women about to get married will be determined in a quantitative study with a two-group quasi-experimental method.

**Discussion:**

Providing a comprehensive and practical intervention program to promote sexual competence based on cultural, social, and religious background can help to improve the quality of sexual interactions of young women about to get married, reduce harm caused by lack of sexual competence, and ensure women’s sexual health.

## Plain language summary

Preparing young people to start safe and satisfactory sexual behaviors and as a result to acquire sexual competence can play an important role in ensuring their sexual health. But in some countries, talking about sexual issues is a cultural and religious taboo. Given that Lack of sexual competence is associated with negative consequences, the present study aims to design, implement, and determine the effectiveness of a program to promote sexual competence in young adult women who are about to get married. In the first phase, to identify the needs of acquiring sexual competence and to explore the strategies for improving sexual competence, a qualitative study will be conducted with a content analysis approach. Individual interviews will be conducted with women aged 18 to 25 about to get married, the teachers of pre-marriage classes, midwives, gynecologists, reproductive health specialists, psychiatrists, and psychologists who work in the field of sexual health. Based on the findings of this phase and after reviewing the literature, an intervention program to improve sexual competence in young adult women will be designed and prepared. After the experts approve the intervention program, in the second phase, a quantitative study will be conducted to determine the effect of the intervention program on improving the sexual competence of young adult women about to get married.

## Introduction

According to the definition of the World Health Organization (WHO, 2006): sexual health is “…a state of physical, emotional, mental and social well-being in relation to sexuality; it is not merely the absence of disease, dysfunction or infirmity. Sexual health requires a positive and respectful approach to sexuality and sexual relationships, as well as the possibility of having pleasurable and safe sexual experiences, free of coercion, discrimination and violence.” [1] To achieve sexual health, it is necessary to acquire competencies in starting sexual interactions.Sexual competence is one of the important dimensions of sexual well-being or health [2] and having it in sexual interactions is one of the challenges of sexual health in any society. Sexual competence means the ability to have sex along with successful processes such as successful interaction between two sexual partners to have sex (no coercion) and negotiation to choose a prevention method and successful results such as prevention of unwanted pregnancy and sexually transmitted diseases [3]. Therefore, if a person in his first heterosexual relationship has the conditions of agreement, autonomy, lack of regret, and the use of reliable contraceptive methods; It is known to have sexual competence [4]. People with high sexual competence are aware of their sexual preferences [2] and can delay sexual interactions and use condoms during intercourse [5]. Also, they behave more responsibly in sexual interactions, which reduces the risk of victimization or sexual assault [6]. On the other hand, the lack of sexual competence can be associated with adverse consequences such as low sexual performance, increased prevalence of sexually transmitted diseases, including increased infection with the human papillomavirus, unwanted pregnancy, and forced sex [7]. These complications for women, in addition to the immediate physical effects, have long-term irreparable consequences such as infertility, pregnancy and childbirth complications, stigmatization, and cancer [8]. In different communities, the health of today’s girls who are tomorrow’s mothers; has an impact on the health of the next generations and the society, and ensuring their health is a form of investment to achieve the goals of sustainable development [9]. But in developing societies, this group struggles with all kinds of gender inequalities, such as preventing education, forced marriage at a young age, and less decision-making power. Several obstacles include political and cultural obstacles that cause the stigma of teaching sexual issues to unmarried girls, and structural problems of health systems that do not include sexual health education for this group. Also, parents’ lack of knowledge and preparation to teach their daughters about sexual issues is an obstacle to acquiring the necessary sexual competence in them [10]. One of the important ways to get the necessary preparations to start healthy sexual interactions is education [11]. Teaching sexual health to people is associated with many benefits, including prevention of violence and sexual abuse, development of healthy relationships, promotion of social/emotional learning, increase of media literacy in sexual matters, and use of condoms [12, 13], which can be presented in a scientific, comprehensive and evidence-based manner formally in schools [14] or informally through friends, family members, peers [15], films [16] and other sources.

**Fig. 1:**
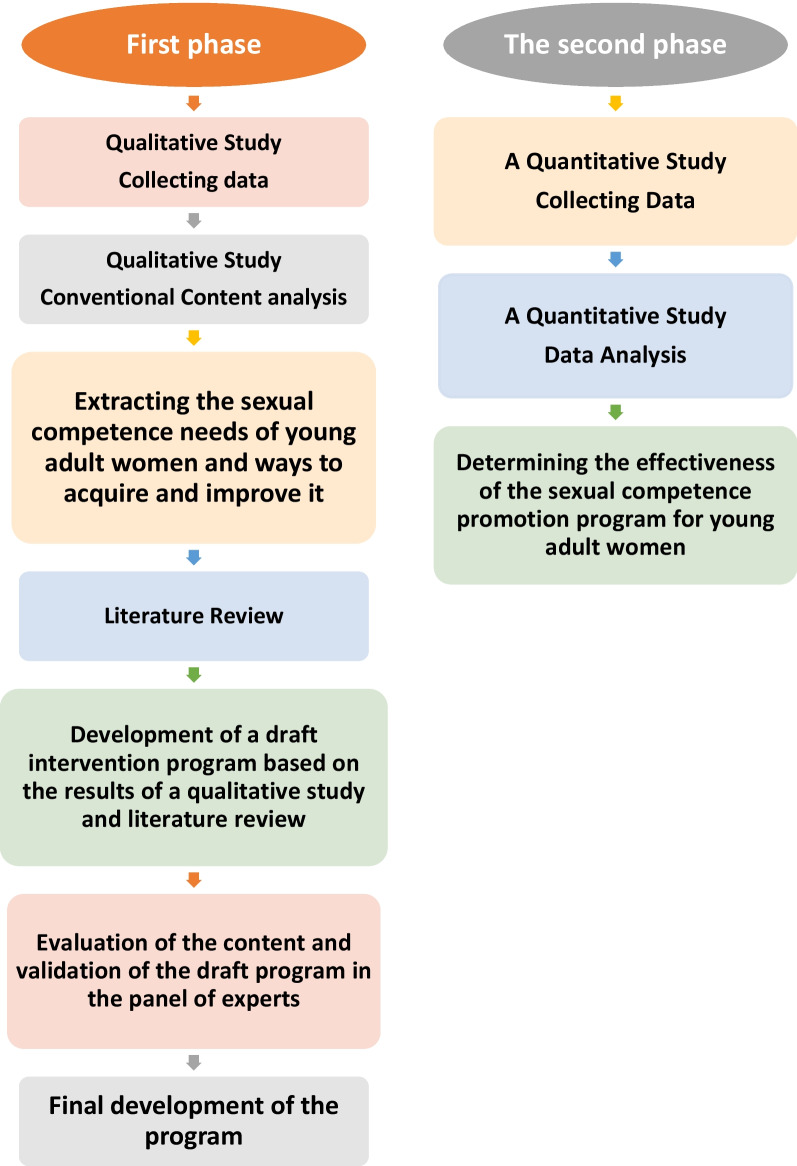
Study steps

In some developing countries, there is no formal sex education for young people, and there is a lot of cultural resistance, especially in Asian countries, against sex education. Traditionally, parents think that sex education to students causes early initiation of sexual activity and increases unsafe sexual behaviors. In these countries, by emphasizing abstinence from sex until marriage, they try to prevent young people from starting sex by instilling fear, guilt, and shame [17]. In most Muslim countries, talking about sexual issues is a cultural and religious taboo [18]. For this reason, formal sex education is not given in schools [19]. However, according to research, sex education in schools not only does not increase harmful sexual behaviors, but also causes delays in the initiation of sexual intercourse, and safe sexual behaviors, increases the use of contraceptive methods, and reduces unwanted pregnancies [20–22] (Fig. 1).

Because sex education programs should be provided based on the culture of each society [23]; In Iran, as a Muslim country, since 1993, the sex education program at the time of marriage was considered for two hours; But these courses are not comprehensive enough, and the level of knowledge and attitude of the participants in the pre-marriage classes about sexually transmitted diseases, sexual health and fertility has been evaluated as weak to moderate [24]. Recently, despite the increase in pre-marriage education [25], the share of sexual health education remains the same two hours and no new content about sexual health education has been added, and it seems that it cannot meet the needs of young people’s sexual competence. On the other hand, in Iran, the legal age of marriage for girls is 13 years [26], and currently, the average age of marriage for girls is 23.4 years [27], and considering that sexual education for children under the age of 18 in Iran, like many Muslim countries, faces limitations; Therefore, the age gap of 18–25 years (young adult) will be a good opportunity to prepare young people to make safe, informed and voluntary reproductive and sexual decisions [28].

Therefore, since there is no formal sex education for young people in Muslim countries, pre-marriage education does not seem to be enough, and the socio-cultural background of Muslim countries prevents young women from acquiring sexual competence; The present study aims to design, implement, and determine the effectiveness of an intervention program to promote sexual competence in young adult women who are about to get married, so that in this way it is possible to propose effective interventions based on the context to improve the sexual competence of this group.

## Materials and methods

### Study design and setting

This research is a mixed-method study of sequential exploratory type (Qual-quan) which will be conducted in two phases. At the beginning of the first phase, a qualitative study will be conducted to explore the needs to acquire sexual competence in young adult women and discover ways to improve it. Based on the findings of this study and after reviewing the literature, an intervention program to improve sexual competence in young adult women will be designed and prepared. After the experts approve the intervention program, in the second phase, a quantitative study will be conducted to determine the effect of the intervention program on improving the sexual competence of young adult women about to get married.

The first phase: This phase includes the steps of qualitative study, literature review, and development of an intervention program to improve the sexual competence of young adult women, which is as follows:

### First step: a qualitative study

In this phase, to identify the needs of acquiring sexual competence and to explore the strategies for improving sexual competence, a qualitative study will be conducted with a content analysis approach.

### Study participants and sampling method

The participants in this phase of the study are young adult women aged 18 to 25 who go to counseling centers, clinics, and health centers to receive health services and pre-marital education. Also, the teachers of pre-marriage classes, midwives, gynecologists, reproductive health specialists, psychiatrists, and psychologists who work in the field of sexual health will be participants. For young adult women about to get married, Purposive sampling will be with maximum diversity in terms of characteristics such as age, occupation, level of education, economic status, way of getting to know the spouse, and birth rank (the number of children in the family). The selection of other participants in the research will also start in a Purposive manner and will continue with the strategy of maximum diversity in terms of work experience.

### Inclusion criteria for adult women about to get married are

Informed consent to participate in the study, ability to communicate and interview, no history of attending pre-marriage classes, young adult women about to get married for the first time.

### Inclusion criteria for other contributors include

Informed consent to participate in the study, teaching experience in pre-marriage classes for more than 2 years (about pre-marriage class teachers).

### Data collection technique

To collect data, in-depth individual interviews will be conducted in a semi-structured way. At first, the researcher introduces herself to the participants and establishes a friendly relationship with the people, the objectives of the research will be explained and they are assured of the complete confidentiality of their answers. Written informed consent will also be obtained. The location of the interview will be determined according to the wishes of the people, and then the interviews will begin using guide questions (Table 1).

**Table 1:** An example of interview guide questions

Participants	Sample questions
Young adult women	What are the requirements for a satisfying sex relationship?
	What do you think safe sex looks like?
Teachers of pre-marriage classes, midwives, gynecologists, reproductive health specialists, psychiatrists and, psychologists who work in the field of sexual health	In your opinion, what is the state of sexual competence of young adult women about to get married?
	What strategies do you suggest to improve sexual competence in young adult women who are about to get married?

To deeply analyze the data, the interview sessions are recorded. After the analysis, if needed, additional interviews will be conducted with the participants or other people who can have more key information related to the phenomenon under study. Immediately after each session, all recorded conversations are written in full. In this research, taking notes in the field will also be used to collect data, for this purpose, the researcher will pay attention to the non-verbal behaviors of the participants and their attitudes and interactions during the interviews and will note them down.

Sampling continues gradually until data saturation is reached. In other words, until no more new information is obtained during data analysis and coding (saturation); Sampling will continue.

### Data analysis

To analyze the data, the conventional content analysis method with the approach proposed by Graneheim and Lundman will be used. After each interview, the recorded interview is transcribed verbatim. Then, by repeatedly reading all the data, the researcher will get an overview that leads to the formation of a semantic unit. In the next step, the semantic units are converted into a more concise and dense form, and at this time, the words that contain the key concepts are highlighted, and thus the codes will be extracted. Then, different codes are placed in sub-categories based on their relationship with each other, and these sub-categories form category based on similarities and differences with each other. Finally, the category will form the main category based on their similarities and differences with each other [29]. MAXQDA 10 software will be used for better data management.

### Trustworthiness of qualitative data

To ensure the accuracy of the research findings, four criteria will be used (credibility, dependability, conformability, and transferability) [30]. To ensure the credibility of the study, in-depth interviews at different times and places, selection of participants with maximum variety, peer debriefing and, member check will be used. To ensure the dependability of the findings, all the activities and steps of the work will be described in detail. Also, the external observer will be used to check his/her possible similar understanding with the researchers and search for discrepancies. To confirm the transferability, the research results will be presented to four people with the profiles of the participants who did not participate in the research. For conformability, the extracted codes will be given to several faculty members who are familiar with the method of qualitative research analysis and did not participate in the research team, so that the correctness of the coding and class extraction process can be checked.

### Second step: literature review

At this stage, the researcher will review the texts using the matrix method to review the existing knowledge in the field of sexual competence needs, strategies, programs and interventions to promote sexual competence and to confirm and complete the needs discovered in the qualitative phase. Search in Persian electronic databases: SID, Magiran, and English databases: ProQuest, Web of Sciences, PubMed, Cochrane Library, Scopus, using the keywords “program” AND “Pre-marriage classes” OR “sexual competence” OR “first sex” AND “young adult” will be done in the period between 2000 and 2023. Also, to complete the search, international guidelines, sex education books and contents of world-reputable sites such as UNESCO, World Health Organization, etc. will also be reviewed.

*Inclusion criteria include:* full text of studies with quantitative, qualitative, and mixed methods, book review and review of texts in Farsi and English, articles published in the period from 2000 to 2023. *Exclusion criteria include:* inappropriate content and insufficient data of articles, articles presented in conferences, abstracts and letters to editors.

Third step: Development of an intervention program to improve the sexual competence of young adult women.

At this stage, using the results of the qualitative study and literature review, a draft of the intervention program to improve the sexual competence of young adult women who are about to get married will be developed. Finally, to check the content of the intervention program and validate it, a panel of experts will be held at Isfahan Nursing and Midwifery College. After reviewing the draft program in the panel of experts, if necessary, changes will be made to it according to the experts’ opinions and the intervention program will be finalized for implementation in the next phase. The members of the expert panel will be 15 people and include pre-marital class teachers, midwives, gynecologists, reproductive health specialists, psychiatrists, and psychologists who work in the field of sexual health. Inclusion criteria for expert panel members are having experience and expertise in sexual health, teaching experience in pre-marriage classes for more than 2 years (about pre-marriage class teachers), and willingness to attend panel meetings.

### Phase II: quantitative study

The aim of the quantitative study is to determine the impact of the intervention program on the sexual competence of young adult women about to get married. This study is a semi-experimental two-group and three-stage study (before, immediately and, 4 months after the intervention) that will be implemented in premarital counseling centers and selected health centers in Isfahan.

### Study participants

It will include young adult women about to get married.

Inclusion criteria include: willingness to participate in the research, age between 18 and 25 years, Iranian nationality, and exclusion criteria include: unwillingness to continue cooperation at any stage of the research and incomplete questionnaires.

### Data collection method

At this stage, by referring to premarital counseling centers and health centers, the samples with the Inclusion criteria are selected for the available sampling method. Before the implementation of the program, written informed consent will be obtained from the samples, and then the allocation of the samples into two control and intervention groups will be done by randomization.

### Sample size

The sample size was calculated according to (α = 0.05, β = 0.1, SD1 = 8.5, and SD2 = 19.8), 37 people in each group; However, due to increasing the validity of the study and considering at least 20% dropout, the number of samples will be 110 people (55 people in the intervention group and 55 people in the control group).

### Methods

The program designed in the previous phase will be implemented for young adult women about to get married in the intervention group. For members of the control group the intervention program will not be implemented; At the end of the study, the content of the sexual competence promotion program will be presented. To evaluate the improvement of sexual competence, the researcher-made questionnaire of sexual competence of young adult women, whose validity and reliability will be determined before the start of the study, will be used in two intervention and control groups before, immediately, and 4 months after the intervention.

### Data analysis

Statistical analysis will be done at two descriptive and inferential levels. At the descriptive level, the mean and standard deviation indicators will be used, and at the inferential level, the Variance analysis model with 2 × 3 repeated measures will be used. Statistical analysis is done using SPSS statistical software version 22 and, in all tests, a maximum error of 5% is accepted.

### Ethical considerations

This study has been approved by the Ethical Committee of Isfahan University of Medical Sciences. (IR.MUI.NUREMA.REC.1402.160). The researcher will obtain informed consent from the participants for each step of the research. If the participants do not want to continue the study, they can withdraw from the study. In the literature review phase, the principles of literary rights will be observed and the most accurate translation of the contents into Farsi will be done along with citing the sources.

## Discussion

Preparing young people to start safe and satisfactory sexual behaviors and as a result to acquire sexual competence can play an important role in ensuring their sexual health. Lack of sexual competence is associated with negative consequences, including increased risk of sexual assault and sexual victimization, negative emotions (such as anger, sadness, fear, and humiliation), increased likelihood of high-risk sexual behaviors (such as not using condoms in sexual relations, drug use) and alcohol before sex, non-use of contraceptive methods) and decreased sexual satisfaction [31]. Therefore, the first step to reduce these side effects is sexual education. According to the published report on the state of comprehensive sexuality education (CSE) in 2021, in 15% of the 155 countries surveyed, there is no policy or law related to sexuality education [32]; As a result, girls without sexual competence face more bad consequences in this field. Because in many societies talking about sexual issues under the age of 18 faces many political, legal, cultural, and religious obstacles; time of marriage can be a good opportunity to receive this training and acquire sexual competence among young people who have been deprived of receiving scientific, up-to-date, accurate and complete training. Therefore, adulthood (18–25 years old) is a good opportunity to learn about reproductive and sexual rights and education and to establish the foundations of sexual health. In the current study, the nature of the research questions is such that first, the status of sexual competence of young adults about to get married should be explained with a qualitative method, then based on the findings and after reviewing the literature, a plan to improve sexual competence should be developed. and its effectiveness will be determined by conducting a quantitative study. Therefore, the best methodology to answer the question of this research is a combined method with a sequential exploratory approach. The sequential exploratory approach is a well-known method of conducting research, especially when little information is available on the topic under study. It is also a good way to get participants’ experiences. Actually, when a research method is not enough to reveal the subject of study, it is better to use a combination of both quantitative and qualitative methods [33].

In most of the research conducted on adolescents and young people, other aspects of sexual life such as sexual experiences [34], sexual adaptation [35], and sexual violence [36] have been addressed; As a result, research on the needs of women’s sexual competence at the time of marriage and ways to improve it can be an effective step in developing and improving the content of sex education before marriage. The results of this study can be used by teachers of pre-marriage classes, midwives, gynecologists, reproductive health specialists, psychiatrists, and psychologists who work in the field of sexual health.

## Data Availability

The datasets used and/or analyzed during the current study are available from the corresponding author on reasonable request.
